# Some like it hot: Thermal tolerance and oxygen supply capacity in two eurythermal crustaceans

**DOI:** 10.1038/srep10743

**Published:** 2015-06-01

**Authors:** Rasmus Ern, Do Thi Thanh Huong, Nguyen Thanh Phuong, Peter Teglberg Madsen, Tobias Wang, Mark Bayley

**Affiliations:** 1 Department of Bioscience, Aarhus University, Aarhus, Denmark; 2 College of Aquaculture and Fisheries, Can Tho University, Can Tho, Vietnam

## Abstract

Thermal sensitivity of the cardiorespiratory oxygen supply capacity has been proposed as the cardinal link underlying the upper boundary of the temperature niche in aquatic ectotherms. Here we examined the evidence for this link in two eurythermal decapods, the Giant tiger shrimp (*Penaeus monodon*) and the European crayfish (*Astacus astacus*). We found that both species have a temperature resistant cardiorespiratory system, capable of maintaining oxygen delivery up to their upper critical temperature (Tcrit). In neither species was Tcrit reduced in hypoxia (60% air saturation) and both species showed an exponential increase in heart and gill ventilation rates up to their Tcrit. Further, failure of action potential conduction in preparations of *A. astacus* motor neurons coincided with Tcrit, indicating that compromised nervous function may provide the underlying determinant for Tcrit rather than oxygen delivery. At high temperatures, absolute aerobic scope was maintained in *P. monodon*, but reduced in *A. astacus*. However, *A. astacus* also displayed reduced exercise intensity indicating that impaired muscle performance with resulting reduced tissue oxygen demand may explain the reduced scope rather than insufficient oxygen supply capacity. This interpretation agrees with early literature on aquatic ectotherms, correlating loss of nervous function with impaired locomotion as temperatures approach Tcrit.

Due to the high thermal conductivity of water and the fundamental effects of temperature on the rates of metabolic processes^1^, water temperature influences physiological rates in all aquatic ectotherms including crustaceans^2^. In general, the effects of temperature on ectotherms are thought to be hierarchical, such that the temperature window for survival is considerably wider than for activity, while the thermal windows for growth and reproduction are narrower^3^,^4^,^5^,^6^. Given the current concerns over the ecological consequences of global warming^7^, considerable efforts are being devoted to identifying and understanding the physiological functions underlying thermal tolerance in aquatic animals.

One model in particular, the “Oxygen and capacity limited thermal tolerance (OCLTT) model”, has gained wide acceptance^7^,^8^ and is currently used in forecasting the effects of climate change on species distributions and future performance^7^,^9^,^10^. This model, based on the work of Fry and Hart^11^,^12^, proposes that cardiorespiratory system failure is the principal determinant of both upper critical temperature where animals lose equilibrium (Tcrit), and the realized environmental thermal niche^8^. Under the OCLTT framework, this failure at Tcrit occurs because the oxygen requirement of the mitochondria at high temperatures, necessary to meet the increasing ATP demands of the tissue, surpasses the oxygen supply capacity of the cardiovascular system. This renders the organism dependent on the much less efficient fermentation pathways for ATP production, and the animal experiences a dramatic loss of performance as energy balance becomes unsustainable.

The majority of studies supporting the OCLTT hypothesis have been performed on polar and temperate stenothermal invertebrates, including crustaceans and fish. When approaching Tcrit, these species are reported to reach a ceiling in heart and gill ventilation performance that limits their ability to increase maximum oxygen uptake rate (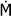
O_2_max) above standard metabolic rate (SMR), resulting in falling blood oxygen levels and a transition to anaerobic metabolism^13^,^14^,^15^,^16^,^17^,^18^,^19^. However, recent studies in a number of eurythermal temperate and tropical species have demonstrated that the cardiorespiratory system (e.g., heart and gill ventilation rates) is able to increase oxygen supply proportionally with tissue oxygen demand as temperature increases to Tcrit. Hence, in these species the excess capacity for oxygen uptake beyond the SMR requirement (absolute aerobic scope), is not significantly reduced approaching the Tcrit. These animals thus avoid the transition to anaerobic metabolism^20^,^21^,^22^,^23^,^24^,^25^,^26^, indicating that factors other than inadequate oxygen delivery must be involved in any loss of ecological performance at high temperature. For example, growth rate in the tropical Giant freshwater shrimp (*Macrobrachium rosenbergii*) is reduced at a temperature below that impacting absolute aerobic scope^24^. It has been proposed, therefore, that eurythermal species have been evolutionarily selected for a thermally resistant cardiorespiratory system^20^,^24^. Thus, it is argued, the OCLTT model fails to explain thermal effects on species living in environments where they frequently encounter unpredictably high temperatures, such as the tropical *M. rosenbergii*^24^ and the intertidal Green crab (*Carcinus maenas*)^20^. In such high temperature or unpredictable environments, there may have been a strong evolutionary pressure to scale the cardiorespiratory system for oxygen delivery right up to Tcrit and thus other physiological parameters must account for species thermal tolerance.

Since it follows from the OCLTT model that the upper thermal limit should be reduced during hypoxia, we tested the model’s universality by measuring Tcrit in normoxia and hypoxia in two eurythermal crustacean species; the tropical Giant tiger shrimp (*Penaeus monodon*) and temperate European crayfish (*Astacus astacus*). Furthermore, we investigated the association between Tcrit and loss of oxygen delivery by quantifying absolute aerobic scope, heart rate and ventilation rate during temperature elevation. Finally, since early literature on the physiological mechanisms underlying Tcrit in aquatic ectotherms suggested that compromised neural activity may be a critical factor for thermal tolerance 27,28,29,30, we measured the impact of elevating temperature on the ability of nerve preparations from *A. astacus* to conduct action potentials.

## Results

### Oxygen uptake

**Figure 1:**
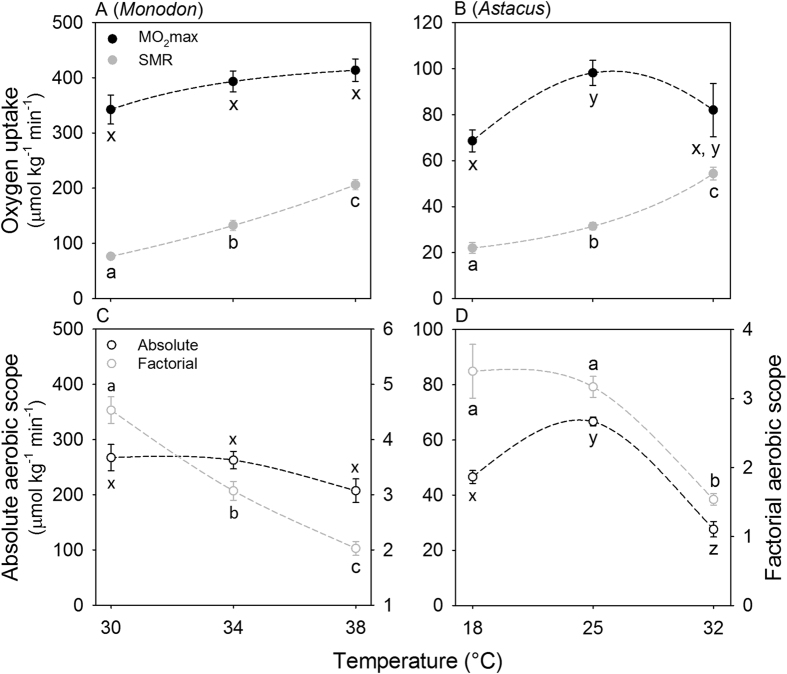
Standard metabolic rate (SMR, closed grey circles) and maximum oxygen uptake rate (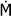
O_2_max, closed black circles) (A, B), and absolute aerobic scope (open black circles) and factorial aerobic scope (open grey circles) (C, D) in the Giant tiger shrimp (*Penaeus monodon*) (n = 8) (A, C) and the European crayfish (*Astacus astacus*) (n = 8) (B, D). SMR in both species were fitted with an exponential growth function and 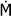
O_2_max with an exponential rise and a Gaussian peak function in *P. Monodon* and *A. Astacus*, respectively. Different letters indicate a significant difference (P < 0.05). Values are means ± SEM.

In *P. monodon* (n = 8), SMR increased exponentially from 30 to 38 °C (P = <0.001), while 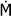
O_2_max was maintained (P = 0.086) within the same temperature interval (Fig. 1A), resulting in a higher Q_10_ for SMR than for 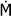
O_2_max (3.5 ± 0.2 and 1.3 ± 0.2, respectively). As a consequence, absolute aerobic scope (i.e. 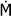
O_2_max – SMR) was maintained (P = 0.063) as temperature rose from 30 to 38 °C and at 38 °C, 2–3 °C below Tcrit, *P. monodon* still retained 72% of the absolute aerobic scope that was available at 30 °C. Factorial aerobic scope (*i.e.* 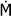
O_2_max / SMR), on the other hand, decreased significantly (P = <0.001) from 30 to 38 °C, with only 45% of the factorial aerobic scope available at 30 °C retained at 38 °C (Fig. 1C). In *A. astacus* (n = 8), SMR increased exponentially (Q_10_ = 1.9 ± 0.1) from 18 to 32 °C (P <0.001), while 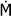
O_2_max increased significantly from 18 to 25 °C (P = 0.044) and plateaued from 25 to 32 °C (P = 0.298) (Fig. 1B). At each temperature, absolute aerobic scope of animals used in the SMR measurements, was calculated by subtracting SMR of individual animals from the mean of 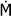
O_2max_, and factorial aerobic scope was calculated by dividing the mean of 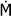
O_2max_ with SMR of individual animals. Absolute aerobic scope increased significantly from 18 to 25 °C (P < 0.001) followed by a significant decrease from 25 to 32 °C (P < 0.001). At this temperature, 2–3 °C below Tcrit, the animals retained 43% of their absolute aerobic scope measured at 25 °C. Factorial aerobic scope was maintained from 18 to 25 °C (P = 0.461) followed by a significant decrease from 25 to 32 °C (P < 0.001), with 49% of the factorial aerobic scope at 18 °C retained at 32 °C (Fig. 1D).

In both species, 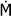
O_2max_ usually occurred within 3–5 min after the exercise protocol and declined gradually to ~50% of 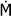
O_2max_ towards the end of the measuring period (10 and 20 min for *A. astacus* and *P. monodon*, respectively). Preliminary measurements over 1 h showed a continued decline in 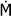
O_2_ without any delayed peaks in 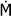
O_2_max. During exercise, the time until exhaustion ranged from 3–5 min at lower temperatures to 2–3 min at higher temperatures. Furthermore, both species became sluggish at higher temperatures and exhibited a reduced number of escape responses prior to exhaustion. This was especially pronounced in *A. astacus* where most individuals were completely unresponsive when nudged at 32 °C.

### Heart rate and gill ventilation rate in normoxia

**Figure 2:**
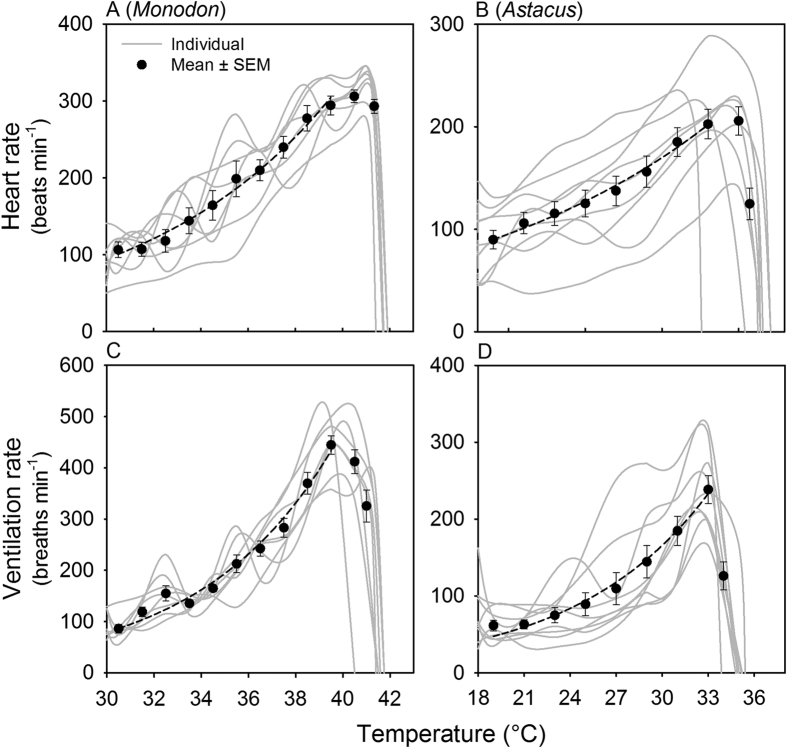
Effects of acute temperature increase (2 °C h^–1^) on heart rate (A, B) and gill ventilation rate (C, D) in resting giant tiger shrimp (*Penaeus monodon*) (n = 8) (A, C) and European crayfish (*Astacus astacus*) (n = 9) (B, D). Data for each species were fitted with an exponential function. In both species, mean heart and gill ventilation rates increased exponentially with temperature up to Tcrit. *P. monodon:* heart: r^2^ = 0.98, Q_10_ = 3.1; gill: r^2^ = 0.93, Q_10_ = 5.0, from 30 to 39.5 °C; *A. astacus:* heart: r^2^ = 0.98, Q_10_ = 1.8; gill: r^2^ = 0.97, Q_10_ = 2.6, from 18 to 33 °C. Grey lines show traces for individual animals, mean ± SEM are shown in black.

Each heartbeat in *P. monodon* (n = 8) and *A. Astacus* (n = 9) provided a single oscillation in the recorded signal. Each gill ventilation cycle consisted of a short oscillation followed by a longer oscillation, corresponding to the elevation and depression of the scaphognathite^24^. The heart and gill ventilation rates of both species increased exponentially with rising temperature until 2–3 °C below Tcrit, where both rates declines declined abruptly, accompanied by loss of buoyancy as the animals became clearly moribund (Fig. 2).

### Critical temperatures in normoxia and hypoxi

**Figure 3:**
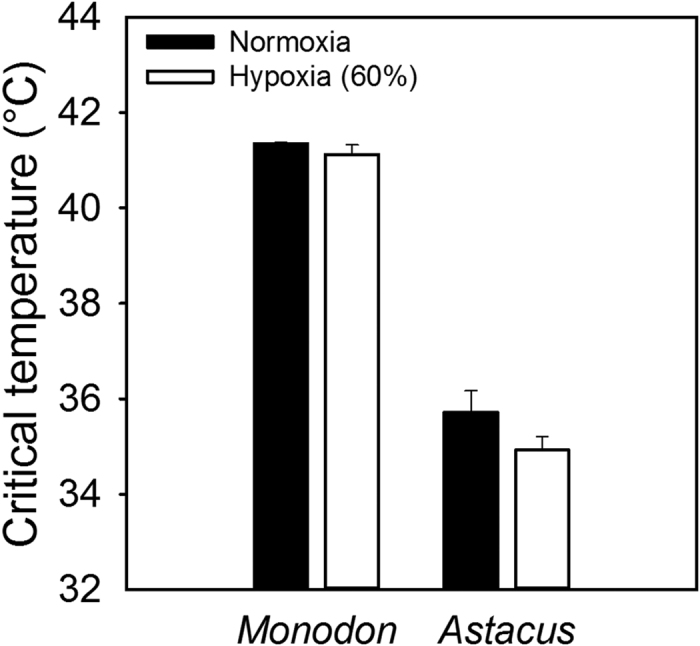
Tcrit (°C) was unaffected by reduced oxygen tension (Normoxia and 60% air saturation) in the Giant tiger shrimp (*Penaeus Monodon*) (n = 8) (P = 0.798) and the European crayfish (*Astacus astacus*) (n = 9) (P = 0.052). Columns show means ± SEM.

Tcrit in normoxia was calculated based on the heart rate measurements in normoxia described above. Heart rate measurements in hypoxia were used solely to calculate Tcrit in hypoxia. Hypoxia (60% air saturation) had no significant impact on Tcrit compared to normoxia (100% air saturation) in either species. Thus, in *P. monodon* Tcrit was 41.3 ± 0.1 and 41.1 ± 0.2 °C (P = 0.798, n = 8) and in *A. astacus* 35.7 ± 0.5 °C and 34.9 ± 0.3 °C (P = 0.052; n = 9) in normoxia and hypoxia, respectively (Fig. 3).

### Action potentials in motor neurons

**Figure 4:**
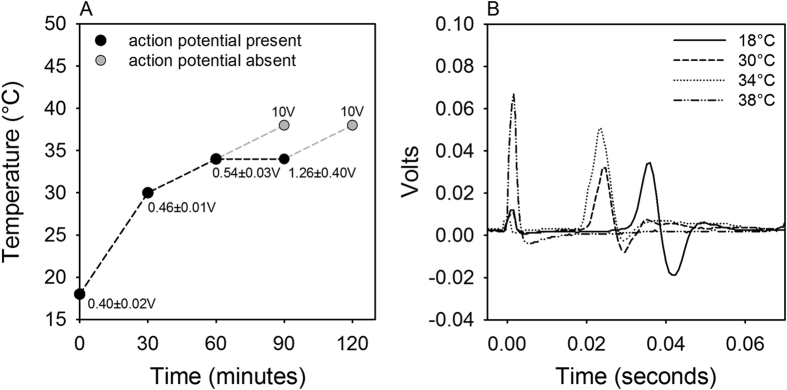
**A**) Effect of time (min) and temperature (°C) on the ability of motor neurons from the legs of European crayfish (*Astacus astacus*) to conduct action potentials (n = 6). The stimulation voltage (± SEM) required to evoke an action potential is indicated at each step. **B**) Derived compound action potentials at 18, 30 and 34 °C in nerve preparations from legs of A. astacus. At 38 °C no action potentials were recorded. At time 0 stimulus artifacts from each recording are seen spikes of different amplitude depending on the stimulation voltage at each step.

The conpduction speed of the action potentials (APs) in motor neurons from *A. astacus* (n = 6) exhibited a Q_10_ of approximately 1.5 in the temperature interval between 18–34 °C. The stimulation voltage required to evoke an AP increased slightly from 0.40 ± 0.02 V at 18 °C to 0.54 ± 0.03 V at 34 °C and more than doubled to 1.26 ± 0.35 V after 60–90 min at 34 °C, indicating a faster deterioration of this *in vitro* preparation at increased temperatures. The nerve bundles maintained at 34 ^o^C for between 60 and 90 min continued to conduct APs, while an increase to 38 °C, at either 60 or 90 min, resulted in an irreversible loss of nervous function, even when the stimulation voltage was subsequently increased by more than an order of magnitude from 0.5 to 10 V. Subsequent temperature reduction from 38 to 30 °C did not restore nervous function (Fig. 4).

## Discussion

In both the tropical *P. monodon* and the temperate *A. astacus*, heart and ventilation rates increased exponentially until temperatures rose to immediately below their respective Tcrit’s (Fig. 2). That their ability to maintain oxygen supply at high temperatures is intact, was further supported by the limited loss of aerobic scope (Fig. 1) and the lack of an effect of hypoxia on Tcrit (Fig. 3). In *P. monodon,* the 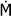
O_2_max was still increasing at the highest measurement temperature, 2–3 °C below the Tcrit. Hence, this species did not show the temperature range of diminishing performance close to Tcrit predicted by the OCLTT model. *A. astacus,* on the other hand, appeared to provide some support for the OCLTT model with a significant reduction in 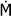
O_2_max between 25 and 32 °C (Fig. 1). However, reduced exercise intensity during 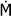
O_2_max measurements coincided with loss of nervous function in *A. astacus* at high temperatures. This impaired muscle performance with associated reduced tissue oxygen demand thus appears to provide a more parsimonious explanation for the reduced 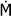
O_2_max than the loss of oxygen supply capacity suggested by the OCLTT model. Both of the species tested here, therefore challenge the universality of the OCLTT model.

The determination of Tcrit during hypoxia represents a direct test of the OCLTT model. According to the model, hypoxia causes a reduced water to blood branchial PO_2_ gradient and hence lower oxygen concentrations in the hemolymph returning to the heart and general circulation^31^. Thus, although crustaceans elevate ventilation and gill perfusion to maintain SMR in hypoxia^31^,^32^, the OCLTT model implies that these cardiorespiratory responses would already be maximized at Tcrit in normoxia, leaving no extra capacity for increasing 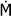
O_2_ from the water. If the model is correct, hypoxia must therefore give rise to a reduced Tcrit. This prediction of the OCLTT model was not born out in the present study, with neither species showing a significant reduction in Tcrit in 60% hypoxia compared to normoxia (Fig. 3). At constant temperatures, crustaceans have been shown to maintain arterial hemolymph oxygen saturation below 60% air saturation^33^. Assuming animals can also maintain oxygen saturation with rising temperatures, this might be interpreted to mean that our hypoxia level was insufficient to challenge oxygen supply. There are no data on the effects of hypoxia on arterial hemolymph oxygen tension in crustaceans approaching Tcrit. However, studies performed during normoxia show decreasing values of both arterial and venous hemolymph oxygen tensions^19^,^34^,^35^,^36^,^37^, indicating that as temperature rises the increasing tissue oxygen demand reduces the oxygen tension of hemolymph returning to the gills, preventing complete oxygen saturation of hemolymph leaving the gills. A 60% air saturation level should therefore present a valid challenge to the oxygen supply capacity in *P. monodon* and *A. astacus*.

Our measurements of the cardiorespiratory responses to acute temperature elevations also indicate an adequate oxygen delivery at temperatures immediately below Tcrit. This is evident in the exponentially increasing heart rate and gill ventilation rates, measured up to temperatures immediately below Tcrit (Fig. 2). Again, this finding is not consistent with the explicit predictions of the OCLTT model^19^. Reduced cardiac stroke volume or tidal volume might potentially have reduced the overall oxygen supply capacity of the cardiorespiratory system. However, the SMR in both species rose exponentially (Fig. 1) with Q_10_-values within the range reported for other crustaceans^38^,^39^,^40^, indicating that oxygen delivery was sufficient to meet the rise in SMR.

Motor neuron bundles excised from *A. astacus* lost the ability to conduct APs at temperatures between 34 and 38 °C (Fig. 4), showing that compromised nervous function correlates with upper thermal limits. Similar conclusions were drawn in earlier studies on the physiological mechanisms underlying critical temperatures in aquatic ectotherms. In goldfish, localized heating of the brain induced the same sequence of behavioral malfunctions as those observed in whole animals during warming, and the decline in spontaneous activity of interneurons within the cerebellum occurred at similar temperatures to those disturbing behaviour^27^. Further, reduced spontaneous activity and behavioral disturbances were linked to thermally induced changes in the viscosity of synaptic membranes via changes in membrane phospholipid saturation^30^,^41^. In the crayfish (*Astacus fluviatilis*), the spontaneous spike activity of isolated neuron bundles increased from 10 to 28 °C, but decreased rapidly above 30 °C^42^,^43^. In crayfish exhibiting an impaired righting reflex between 26.7 and 30°C, the temperatures for total collapse of spontaneous activity in isolated nerve cords was between 36.4 and 38.7 °C^28^. Our data are consistent with these studies, as *A. astacus* became sluggish and unresponsive at 32 °C and lost the ability to conduct action potentials above 34 °C, a few degrees below Tcrit (36.2 ± 0.4 °C). As an alternative explanation to the OCLTT hypothesis, high temperatures may disrupt passive membrane ion permeabilities for Na^+^ and K^+^ altering cell excitability, as was the proposed explanation for early heat death in the crayfish (*Astacus pallipes*) at 35 °C^44^. Further, in muscle tissue, the breakdown of passive membrane permeability has been linked to temperature-induced inactivation of Mg^2+^-ATPase in the sarcolemma^29^. These membrane permeability disruptions can result in collapse of normal trans-membrane ion gradients, thus disrupting the resting potential and normal bioelectrical properties of excitable cells, leading to a progressive loss of function. Further, loss of nervous function can also result from enzyme denaturation with associated elevations in heat shock protein (HSP) expression. This was reported in the American lobster (*Homarus americanus*) with increased HSP70 levels and impaired locomotion (righting response) at 28 °C and an Arrhenius break temperature in heart rate at 30 °C, indicative of compromised cardiac function^20^ and hence oxygen supply. The neurogenic heart of crustaceans requires central nervous system input for contraction and maintenance of cardiac rhythm, which exerts both inhibitory and excitatory stimulation through the cardiac ganglion^45^,^46^. Heat induced deterioration of nervous functions at Tcrit therefore clearly have the potential to cause heart malfunction and thus loss of oxygen conductance.

Aerobic scope, the capacity to increase 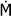
O_2_max above SMR, is widely used to assess the oxygen supply capacity of the cardiorespiratory system^2^,^47^,^48^,^49^. Aerobic scope can be expressed as the absolute increase of 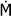
O_2_ above SMR (*i.e.* 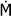
O_2_max - SMR) or as the factorial rise (*i.e.* 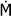
O_2_max / SMR). Calculated with the same data, these two measures can run counter to each other and conclusions based on one value can potentially oppose those based on the other^50^. There is no consensus as to which of these values is most correct, but since there is a direct relationship between sustained work performed and the oxygen required, absolute aerobic scope is arguably a better measure than the proportional rise above baseline metabolism^50^. We therefore base our conclusions on this metric.


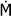
O_2_max is normally measured during, or immediately after intense exercise^50^ but the experimental protocol should reflect the lifestyle of the animal studied. In athletic species capable of prolonged exercise, such as tuna or migrating salmon, a 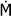
O_2_max measured during continuous swimming is normally used (e.g.^47^). *P. monodon* and *A. astacus*, however, are sluggish, benthic species that primarily reach peak performance during their short, intense and characteristic escape response. The post-exercise 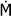
O_2_max protocol was therefore appropriate for these species. It has been hypothesized that in animals where Tcrit is limited by oxygen delivery, 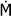
O_2_max will plateau or even decrease at high temperatures as the structural and physiological ceilings for oxygen delivery are reached by their cardiorespiratory systems^8^,^51^. Since SMR increases exponentially with temperature, absolute aerobic scope in these species decreases with rising temperatures and should, according to the OCLTT model^8^, be eliminated at Tcrit. Absolute aerobic scope in *P. monodon* was maintained immediately below Tcrit (Fig. 1C) in support of our hypothesis that eurythermal animals have evolved cardiorespiratory systems capable of delivering oxygen at high temperatures^20^,^22^,^23^,^24^. The interpretation of these data in *A. astacus* is slightly more involved. While its absolute aerobic scope was maintained at lower temperature, this parameter was significantly reduced at high temperatures (Fig. 1D), in accordance with the early findings of Fry and Heart^11^,^12^. At first glance this species seems to provide support to the OCLTT model^8^. However, because the Tcrit was not impacted by hypoxia, but was associated with loss of nerve function, we argue that the slight loss of oxygen supply capacity measured here cannot be the direct cause of the Tcrit, but is rather a secondary correlate. Hence neither of these two eurythermal species provide support for the OCLTT hypothesis.

Although the tail muscle is largely powered by anaerobic ATP production^52^ such that the first escape response is not directly dependent of oxygen availability, both species became sluggish at higher temperatures and exhibited a reduced number of escape responses prior to exhaustion. This was especially pronounced in *A. astacus* where most individuals were completely unresponsive when nudged at 32 °C. The observed sluggishness of both species at high temperatures may result from compromised function of both afferent nerves from sensory systems relaying information about the mechanical stimulus and of efferent motor nerves innervating the muscles. If impaired muscle performance reduces tissue oxygen demand, the lack of responsiveness observed in *A. astacus* at 32 °C may explain the reduced aerobic scope in this species at 32 °C, rather than thermally induced limitations in oxygen supply capacity as suggested by the OCLTT model. If this hypothesis is correct, critical temperatures should not be affected by reduced water oxygen tension, consistent with our observations on both *P. monodon* and *A. astacus* (Fig. 3). If deterioration of nervous function impedes tail muscle tissue performance at 32 °C we might also expect diminished heart and gill movement at this temperature. An explanation for the observed lack of constrained heart and gill ventilation rates at 32 °C in *A. astacus* may be that the nerves innervating in these vital structures are less temperature sensitive than nerves innervating tail muscles. This is of course speculative and provides an interesting topic for future studies.

The “Multiple performances - multiple optima” (MPMO) hypothesis^50^ is an alternative to OCLTT, arguing that physiological functions have different optimal temperatures in the functions underlying animal fitness, and that their relative contribution to fitness varies between species. Our findings with *P. monodon* and *A. astacus* support the MPMO idea, indicating that although some functions may be impacted by oxygen supply capacity, others including those responsible for lethal temperatures appear to be dictated by nervous function. Whether or not thermal tolerance in tracheated arthropods is determined by oxygen limitations has likewise been debated^53^,^54^, and within this very diverse group it is evident that some species have evolved thermally resistant gas exchange mechanisms, while others have not^55^. Thus, while the oxygen supply capacity of the cardiorespiratory system does seem to be coupled to thermal tolerance in some polar and temperate stenothermal species^8^, there is increasing evidence in eurythermal species that this is not the case. These eurythermal animals appear to possess a more thermally resistant cardiorespiratory system^23^,^24^,^26^,^56^, adapted to provide sufficient oxygen supply in unpredictable high temperature environments. Indeed, it would seem unlikely that the upper critical temperature was universally determined by a single cardinal physiological rate across all species. It seems more plausible that failure of any of a variety of physiological systems including the cardiovascular system, the nervous system, enzyme imbalances, membrane fluidity etc., are possible, and that species differences are highly likely. General predictions of the effect of climate change on aquatic animals, based on oxygen supply capacity alone, should therefore be made with great caution.

## Methods

### Animals and maintenance

Giant tiger shrimp (*Penaeus monodon*) were obtained from a hatchery outside Can Tho (Southern Vietnam) and held in 1m^3^ tanks at Can Tho University at 29 ± 1 °C and salinity at 28 ppt. This salinity level matched the hatchery salinity and was reached by mixing dechlorinated tap water with concentrated (100 g L^–1^) seawater from The South China Sea. Water was changed regularly to ensure that NH_4_^+^-N, NO_2_^–^ and NO_3_^–^ concentrations never exceeded 0.25, 0.3 and 20 mg L^−1^, respectively. European crayfish (*Astacus astacus*) were obtained from a hatchery in southern Jutland (Denmark) and kept at Aarhus University (Denmark) in 1 m^3^ freshwater tanks (18 ± 0.1 °C), constantly supplied by particle-filtered, UV sterilized and protein skimmed water. Both species were fed to satiety on dry feed and freshly thawed shrimp every second day. To prevent the metabolic effects of molting and digestion on oxygen uptake rate (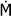
O_2_), all measurements of SMR were performed in the intermolt period after 4 days of fasting. Different animal groups were used for each of the 5 experiments; oxygen uptake, gill ventilation rates in normoxia, heart rates in normoxia, heart rates in hypoxia, and evoked action potentials in motor neurons.

### Oxygen uptake


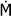
O_2_ in both species was measured using computerized intermittent-flow respirometry^57^,^58^. Perforated Plexiglas plates mounted in each end of the respiration chamber ensured mixing of the circulating water during closed periods. Using equation (1), 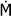
O_2_ was calculated from the decline in PO_2_ during the closed period, with a 2 min delay from the start of the closed period:
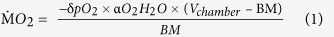
where 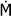
O_2_ is oxygen uptake rate (μmol kg^–1^ min^–1^), δpPO_2_ is slope of the decline in oxygen tension of the water (mmHg min^-1^) when the respirometer is closed, αO_2_H_2_O is the solubility of oxygen in water at the relevant temperature (μmol L^−1^ mmHg^-1^)^59^, V_chamber_ is the volume of the respirometer (L), and BM is body mass (kg). It was assumed that the animal displaced a water volume similar to its body mass (*i.e.*, a density of 1 g ml^−1^).

In *P. monodon,* 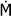
O_2_max and SMR were measured at 30, 34 and 38 °C. At each temperature SMR and 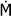
O_2_max were measured in 8 animals, using a total of 24 animals (body mass = 33.9 ± 0.8 g). The animals were placed in the respirometer (1.4 L) at 30 ± 0.1 °C and temperature was either maintained at 30 °C or elevated to the required temperature at 4 °C h^−1^. Upon reaching the target temperature, animals were left undisturbed for 1h before measurement of 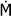
O_2_ for the subsequent 4 h. 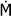
O_2_ measurements consisted of successive 15 min time-loops of 10 min of oxygen consumption measurement and 5 min of respirometer flushing to return the respirometer PO^2^ to normoxia. Having completed SMR measurements, 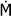
O_2_max was measured by moving the animal to a water tank at the same temperature as the respirometer and inducing vigorous escape responses by nudging the carapace with a soft brush. When the animal became unable to perform an escape response and seemed exhausted, it was returned to the respirometer and 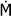
O_2_ measured over the subsequent 10 min.

In *A. astacus*, 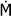
O_2_max and SMR were measured at 18, 25 and 32 °C. SMR was measured in a total of 8 animals; individual animals being measured at all 3 temperatures (body mass = 72.8 ± 3.4 g). The animals were placed in the respirometer (1.4 L) at 18 ± 0.1 °C and left undisturbed for 1 h, after which 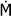
O_2_ was measured over 3 h. Temperature was then elevated at 4 °C h^−1^ to 25 ± 0.1 °C and the animal left for 1 h before 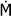
O_2_ was measured again for 3 h. This 1 h delay before measurement was necessary to allow the Hamilton oxygen probes to stabilize at the new temperature for reliable PO_2_ measurements. The same procedure was repeated at each temperature. Each 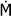
O_2_ measurement comprised 30 min time loops (longer measurement period necessary with *A. astacus* than *P. monodon* because of lower metabolic rate), with a closed period of 20 min followed by 10 min of flushing. 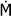
O_2_max was measured separately in 8 animals at each temperature, using a total of 24 animals. Here, the animal was placed in a water tank at 18 ± 0.1 °C and the measurement initiated or the temperature elevated to the target temperature at 2 °C h^−1^. At the target temperature, vigorous escape responses were induced as above until the animal became sluggish, after which it was returned to the respirometer and 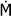
O_2_ measured over the subsequent 20 min.

Both species settled rapidly in the respirometers to produce 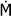
O_2_ traces of great consistency during SMR measurements at each temperature plateau, and the lowest of these consecutive measurements was chosen as the SMR estimate. The decline in PO_2_ during the 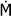
O_2_max measurement was divided into bins of 3 min and the bin (usually the 2^nd^ or 3^rd^) with the highest δPO_2_ used as the 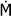
O_2_max estimate. During all measurements, the average decline in water PO_2_ in the respiration chamber was 107 ± 7 mmHg. Randomly distributed blind tests to measure bacterial respiration after the animals had been removed from the respirometers showed that background 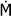
O_2_ due to bacterial respiration never exceeded 5% of SMR.

### Heart and gill ventilation rates in normoxia

Heart and gill ventilation rates in *P. monodon* (n = 8) and *A. astacus* (n = 9) were measured in individual animals using a total of 16 (body mass = 87.9 ± 5.2 g) and 18 (body mass = 85.3 ± 4.4 g) animals, respectively. Heart and gill ventilation rates were measured using reflective infrared sensors (AMP03, Newshift Ltd, Leiria, Portugal) fitted into plastic tubes and glued onto the carapace above either the heart or the scaphognathites. The animal thus instrumented was submerged at 30 ± 0.1 °C (*P. monodon*) or 18 ± 0.1 °C (*A. astacus*) in fully oxygenated water and left overnight to obtain resting values. The following morning, water temperature was elevated linearly at 2 °C h^−1^, while heart or ventilation rates were recorded using Biopac MP100 data acquisition system at 200 Hz (12 bit).

### Critical temperatures in normoxia and hypoxia

Tcrit during both normoxia and hypoxia was determined based on the heart rate measurements described above. Cardiac arrest was seen as a decrease in both heart rate and signal amplitude; resulting in the heart trace gradually leveling out and disappearing as the heart slowed and stopped. To ensure consistency in the critical temperature estimation, the Tcrit was defined as the first 1/6 °C temperature interval where heart rate was in clear decline. While the exact time of arrest was difficult to define, the arrest process was in all cases rapid and occurred within approximately 0.5 °C. Animals were not recoverable and therefore terminated by separating head and body at the end of each measurement. Hypoxia exposure was achieved by submerging the instrumented animal at 30 ± 0.1 °C (*P. monodon*) (n = 8) (body mass = 88.9 ± 4.1 g) or 18 ± 0.1 °C (*A. astacus*) (n = 9) (body mass = 82.1 ± 5.1 g) fully oxygenated water and left overnight. The following morning, water PO_2_ was reduced to 60% air saturation over 1h by bubbling with air and nitrogen mixed in a Wösthoff pump. At 60% air saturation the temperature was elevated linearly at 2 °C h^−1^, while heart rate was recorded. The water PO_2_ was maintained at 60% air saturation during the measuring periods. The duration of the measuring periods was ~6 h for *P. monodon* and ~9 h for *A. astacus*. Heart rate measurements in hypoxia was used solely to calculate Tcrit in hypoxia.

### Measurements of evoked action potentials in motor neurons as a function of temperature

To investigate how temperature influenced nerve function, we measured the ability of nerve preparations to conduct action potentials (APs). Compound, extracellular APs were measured from motor neurons dissected from the legs of the *A. astacus* (n = 6) (body mass = 62.9 ± 8.8g). For each individual animal, two randomly chosen legs were removed and placed in a modified Krebs-Ringer’s solution consisting of 205 mM NaCl, 5.4 mM KCl, 13.5 mM CaCl_2_(H_2_O)_2_, 2.6 mM MgCl_2_(H2O)_6_, and 10 mM Hepes, titrated to pH 7.6 with NaOH^60^ at 18 °C. A small incision was made at each side of the 2nd leg joint and the two halves were separated to expose the central nerve bundle. The exposed nerve bundles were moved to a small bath of temperature controlled Krebs-Ringer solution (18 °C) and suspended between two silver coated electrodes^61^. One end was stimulated extracellularly with a Grass SD9 square pulse stimulator (frequency: 20s^−1^, duration: 0.2 ms, volts: 0.3 V–10 V), while the compound signal was recorded and amplified in the other end with a Grass P55 General Purpose AC Preamplifier (30–3000 Hz band-pass, 100 time amplification) and digitized with a HP 5506 oscilloscope triggered by the stimulator (Grass SD9). The oscilloscope averaged over 8 subsequent stimulations and 2000 data points from each averaged trace were transferred to a PC for further analysis. Temperature was regulated by circulating temperature-controlled water through copper-tubes embedded within the bottom of the bath. The embedded copper–tubes were not in direct contact with the water. The presence or absence of a derived AP was recorded at 18, 30, 34 and 38 °C. Each temperature rise was made over a 10 min period and the nerve bundle was left for 20 min at the new temperature before measurements were repeated. In one of the two nerve bundles from each crab, the presence or absence of a derived AP was measured at four temperatures. To establish whether the nervous function deteriorated at 34 °C, the second nerve bundle was studied at 18 and 30 °C, followed by two measurements at 34 °C performed 30 min apart before the temperature was elevated to 38 °C. A few drops of Krebs-Ringer solution from the bath were applied regularly to the air-exposed ends of the nerve bundles during the entire experiment to avoid desiccation.

### Statistical analysis

Student’s t-test (P < 0.05) was used to test the effect of water PO_2_ on critical temperatures in *P. monodon* and *A. astacus*, respectively. In *P. Monodon* a One-Way Analysis of Variance (P < 0.05) was used to test the effect of temperature on SMR, 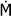
O_2_max, absolute and factorial aerobic scope, respectively. In *A. astacus*, a One-Way Analysis of Variance (P < 0.05) was used to test the effect of temperature on 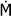
O_2_max and a One-Way Repeated Measures Analysis of Variance (P < 0.05) used to test the effect of temperature on SMR, absolute and factorial aerobic scopes, respectively. Statistical analyses were conducted using SigmaPlot (Systat Software, Inc., Chicago, IL, USA).

## Additional Information

**How to cite this article**: Ern, R. *et al.* Some like it hot: Thermal tolerance and oxygen supply capacity in two eurythermal crustaceans. *Sci. Rep.* **5**, 10743; doi: 10.1038/srep10743 (2015).
